# Raman spectroscopy as a non-invasive diagnostic technique for endometriosis

**DOI:** 10.1038/s41598-019-56308-y

**Published:** 2019-12-24

**Authors:** Ugur Parlatan, Medine Tuna Inanc, Bahar Yuksel Ozgor, Engin Oral, Ercan Bastu, Mehmet Burcin Unlu, Gunay Basar

**Affiliations:** 1 Bogazici University, Physics Department, Istanbul, 34470 Turkey; 2 Esenler Maternity and Children’s Hospital Obstetrics and Gynecology Department, Istanbul, 34230 Turkey; 3 Istanbul University Cerrahpasa School of Medicine, Reproductive Endocrinology and Infertility Division, Obstetrics and Gynecology Department, Istanbul, 34301 Turkey; 4 Acibadem University School of Medicine, Department of Obstetrics and Gynecology, Istanbul, 34752 Turkey; 5 Istanbul Technical University, Physics Engineering Department, Istanbul, 34469 Turkey

**Keywords:** Randomized controlled trials, Randomized controlled trials, Raman spectroscopy, Raman spectroscopy, Raman spectroscopy

## Abstract

Endometriosis is a condition in which the endometrium, the layer of tissue that usually covers the inside of the uterus, grows outside the uterus. One of its severe effects is sub-fertility. The exact reason for endometriosis is still unknown and under investigation. Tracking the symptoms is not sufficient for diagnosing the disease. A successful diagnosis can only be made using laparoscopy. During the disease, the amount of some molecules (i.e., proteins, antigens) changes in the blood. Raman spectroscopy provides information about biochemicals without using dyes or external labels. In this study, Raman spectroscopy is used as a non-invasive diagnostic method for endometriosis. The Raman spectra of 94 serum samples acquired from 49 patients and 45 healthy individuals were compared for this study. Principal Component Analysis (PCA), k- Nearest Neighbors (kNN), and Support Vector Machines (SVM) were used in the analysis. According to the results (using 80 measurements for training and 14 measurements for the test set), it was found that kNN-weighted gave the best classification model with sensitivity and specificity values of 80.5% and 89.7%, respectively. Testing the model with unseen data yielded a sensitivity value of 100% and a specificity value of 100%. To the best of our knowledge, this is the first study in which Raman spectroscopy was used in combination with PCA and classification algorithms as a non-invasive method applied on blood sera for the diagnosis of endometriosis.

## Introduction

Endometriosis is defined as the growth of endometrial gland and stroma outside the endometrial cavity, which is caused by an outflow into the peritoneal cavity. Previous reports demonstrated that one in ten women all around the world sought medical support due to endometriosis and endometriosis-related symptoms including pelvic pain (38.7%), dyspareunia (29.5%), and infertility (11.6%)^1^. Given that the diagnosis of endometriosis depends on histopathologic examination after surgical excision, this approach requires anesthesia induction and hospitalization. Therefore, it significantly affects the quality of life of patients. Thus, researchers focus on new non-invasive methods for the diagnosis of endometriosis, including transvaginal ultrasonography, analysis of blood biomarkers, and genetic predispositions.

Raman spectroscopy provides information about molecular structures and chemical bonds of substances via the detection of inelastically scattered photons^2^. In Raman spectroscopy, the sample is illuminated by a laser beam and inelastically scattered light, which is composed of different frequencies, is observed. The scattered light contains two types of scattering, namely Rayleigh and Raman scattering. The intensity of the light in Rayleigh scattering is strong and the frequencies of the scattered and the incident light are the same, whereas in Raman scattering, the intensity is very weak (about 10^−6^ of the incident beam intensity) and the frequency of the scattered light is different from the frequency of the incident light. The difference between the frequencies of Rayleigh scattering and the inelastically scattered photons can be defined as the Raman shift. The Raman shifts correspond to the vibrational frequencies of the molecules in a targeted sample.

The vibrational frequencies of each chemical bond within a molecule (e.g., O-H, C-O) are different, hence their fingerprints can be uniquely seen in the spectrum. It was reported in a study that during the disease, the amount of protein biomarkers in the blood varied, and these variations could be identified using multiplex and single immunologic testing technologies^3^. In this context, Raman spectroscopy is a useful tool for detecting the chemical content of a sample. Biologic samples such as tissue, blood, and serum are well-suited measurement samples for Raman spectroscopy because chemical changes accompany progressions of most diseases. Therefore, Raman spectroscopy has significant potential to provide valuable information to physicians in medical diagnostics^4^.

Studies have shown that disease diagnostics with Raman spectroscopy is possible for both tissue and blood serum samples. Raman spectra of blood serum samples were used to diagnose many types of diseases, including Alzheimer’s disease^5^, oral cancer^6^, nasopharyngeal cancer^7^, colorectal cancer^8^, dengue infection^9^, lung cancer^10^, hepatitis B^11^, and breast cancer^12^. Endometriosis, however, has thus far only been studied using Raman spectroscopy through tissue. Lieber *et al*., indicated that Raman spectroscopy could differentiate tissues diagnosed as normal or endometriotic from tissues that were diagnosed as benign-cystic or cancerous^13^. Patel *et al*. showed that stages of endometrial cancer could be distinguished using Raman imaging^14^. In another study by Notarstefano *et al*., luteinized granulosa cells were measured using Raman micro-spectroscopy to separate ovarian endometriosis from control samples^15^.

Recently, k-Nearest Neighbor (kNN) and Support Vector Machines (SVM) combined with Principal Component Analysis (PCA) have frequently been used together with spectroscopy in disease diagnostics. kNN is a classification method based on the commonality within groups; every single spectrum can be treated as a point in a multidimensional space. This method calculates the Euclidean distance between each pair of spectra points. Then, by regarding the majority vote of its nearest neighbors, the class assignment of a sample is performed^16^.

Support Vector Machine algorithm is a powerful, supervised learning algorithms, which were introduced by Vapnik^17^. It is used as a classification method in which every data element is viewed as a point in n-dimensional space (n is the number of features) with the value of each feature being the value of an individual coordinate. Classification of the data is achieved by determination of the hyperplane that maximizes the margin between the groups. It is an elegant approach for the classification of spectral data^18–21^.

In some recent studies, classification methods and Raman spectroscopy were used together for disease diagnostics. Dingari *et al*. reported that Raman spectroscopy and multivariate classification could discriminate lesions in stereotactic breast biopsies, irrespective of microcalcification status^22^. Li *et al*. developed a method for the non-invasive detection of colon cancer using Raman spectroscopy together with PCA and kNN^23^.

In this article, we report the first Raman spectroscopy-based classification model that can be used as a non-invasive diagnostic technique for endometriosis. This new approach requires only blood serum from a patient with endometriosis for the diagnosis of the disease. Therefore, the diagnosis of endometriosis could be achievable without laparoscopy.

## Results and Discussion

**Figure 1:**
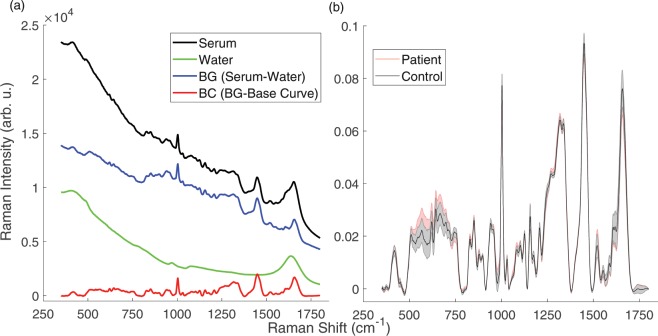
(**a**) Background (BG) and baseline-corrected (BC) Raman spectra of a serum sample. (**b**) Normalized BC mean Raman spectra of the control and patient groups. Standard deviations of each group were plotted and overlaid as shaded curves.

**Figure 2:**
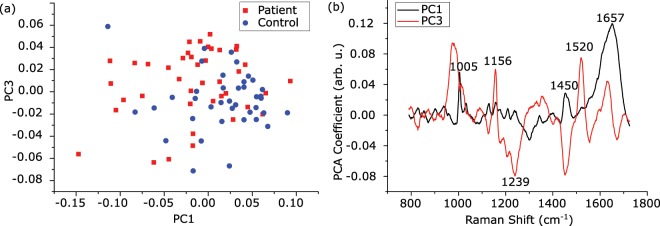
PCA performance on the training data set, which includes normalized BC data from 41 patients and 39 healthy individuals. (**a**) PCA score plot (PC1 vs. PC3) (**b**) Loading 1 and Loading 3 spectra.

The mean Raman spectra of the two groups are demonstrated in Fig. 1b. Although the intensity difference between the groups in the spectral range of 500–750 cm^−1^ is apparent, this spectral interval was not used in the classification processes because the signal variance is high in that region. The appropriate region was chosen for the classification using the variable selection procedure, which is described in the *methods* section. For this procedure, the mean accuracy values of the classification models with the standard deviations (given in parentheses) were calculated and are given in Table 1. The final feature selection was decided by considering the region with the highest mean accuracy value, which was found as 790–1729 cm^−1^ spectral interval. Then, PCA was applied on the normalized and baseline corrected Raman spectral data to extract the relevant features for the selected region (790–1729 cm^−1^). The number of PCs was set in the 95% of the total variance explained (TVE). The percentage TVE values for PCs were calculated as 48.3, 17.2, 13.6, 5.2, 4.3, 2.9, 2.1, and 1.6, respectively. This condition requires 8 PCs for this model. All 8 PCs were included in the model. Figure 2a shows the PCA scores of the first against the third PC to visualize the discrimination of the two groups on the orthogonal feature plane.

**Table 1:** Comparison of the mean accuracy results of kNN and SVM classification models for the four selected regions after 10 repetitions of calculations.

Feature Selection	Mean Accuracy (%)			
Region (cm^−1^)	kNN-f	kNN-w	SVM-c	SVM-q
450–1729	76.2 (2.9)	78.0 (3.6)	73.8 (4.1)	76.9 (4.3)
790–1729	79.4 (3.8)	82.1 (2.5)	80.0 (2.3)	82.5 (2.9)
1140–1729	72.8 (5.1)	77.3 (2.2)	77.5 (3.4)	78.5 (2.2)
1368–1729	63.3 (1.9)	65.8 (4.2)	68.5 (5.8)	64.5 (1.8)

Some of the peaks labeled on the loading graph, given in Fig. 2b, demonstrate shifts and variations, which can be interpreted as changes in structure and the amount of some chemicals in serum during the disease. Among these bands, 1005 cm^−1^ was tentatively assigned to phenyl ring angular vibrations due to phenylalanine content or C=CH bending vibration due to the ground state beta carotene content^24^. The presence of beta carotene also contributed to the 1156 and 1520 cm^−1^ bands, which are C-C and C=C stretching bond vibrations, respectively^25^. The peak at 1450 cm^−1^ was assigned to CH_2_ bending vibration, which exists in lipids, phospholipids, and some amino acids^26^. Besides these, the peaks around 1239 and 1650 cm^−1^ were assigned to amide III (parallel beta sheet) and amide I, respectively. They are related to the secondary structure of proteins such as alpha helix (1657 cm^−1^), parallel *β*-sheet (1630 cm^−1^), and turn (1670 cm^−1^)^12^. The importance of these bands in the diagnosis of endometriosis is not yet clear, and it is to be investigated in the future. On the other hand, the alteration of the bands at 1156 and 1520 cm^−1^ may refer to a change in the amount of beta carotene in the patient group. One explanation for this change could be that the alteration of retinoic acid metabolism in patients with endometriosis^27^. Taylor *et al*. reported a decrease in carotenoids in endometriotic tissues, which may be provide hope for medical therapies as adjuvants or alternatives to the surgical excision^28^. Therefore, beta carotene, which is an important member of the carotenoid family, may have a protective role against endometriosis.

After PCA, the study was carried a step further to examine the performance of the machine learning algorithms on the classification of the Raman spectral data. For this purpose kNN (fine and weighted) and SVM (cubic and quadratic) were used. The data set included measurements from 49 patients and 45 healthy individuals. The training and the cross-validation (5-fold) data sets were separated by selecting 85% of the total data (containing 41 patient and 39 control measurements) randomly. The remaining 15% (including 8 patient and 6 control measurements) of the data was used as unseen data to assess the predictive power of the classification models.

The performance of the applied classification methods in terms of sensitivity, specificity, positive predictive value (PPV), and negative predictive value (NPV) is presented in Table 2. Sensitivity and specificity are measures of classification success in predicting diseased and control specimens, respectively. Detailed explanations of these terms are given in Table 3. The results indicated that application of the kNN-weighted algorithm on the spectral data exhibited the highest classification model accuracy among the others. Using this algorithm in the training procedure, 33 of 41 patients and 35 of 39 control samples were correctly classified. During the testing phase, the model was allowed to guess the correct label (“patient” or “control”) of the unseen datum one by one. The results indicated that the model correctly classified 8 of the 8 patients and 6 of the 6 control samples. In short, this result indicates a promising potential for the use of Raman spectroscopy together with the kNN-w classification algorithm for non-invasive diagnostics of endometriosis.

**Table 2:** Comparison of the predictive ability of kNN and SVM classification models.

Training	kNN-f^(a)^	kNN-w^(b)^	SVM-c^(c)^	SVM-q^(d)^
Specificity	84.6 (33/39)	89.7 (35/39)	84.6 (33/39)	87.1 (34/39)
Sensitivity	78.0 (32/41)	80.5 (33/41)	75.6 (31/41)	75.6 (31/41)
PPV	84.2 (32/38)	89.2 (33/37)	75.6 (31/35)	83.8 (31/37)
NPV	78.6 (33/42)	81.4 (35/43)	83.8 (34/45)	76.7 (33/43)
**Test**	**kNN-f**^**(a)**^	**kNN-w**^**(b)**^	**SVM-c**^**(c)**^	**SVM-q**^**(d)**^
Specificity	100 (6/6)	100 (6/6)	100 (6/6)	100 (6/6)
Sensitivity	87.5 (7/8)	100 (8/8)	87.5 (7/8)	87.5 (7/8)

All results are given in percentages. Information given in parentheses represents the ratio of number of correct predictions to the number of true class measurements.

^(a)^fine, ^(b)^weighted, ^(c)^cubic, ^(d)^quadratic.

**Table 3:** The definitions of sensitivity, specificity, positive predictive value (PPV), negative predictive value (NPV), and accuracy.

	Actual Positive (P)	Actual Negative (N)	
**Predicted Positive**	True Positive (TP)	False Positive (FP)	PPV TP/(TP + FP)
**Predicted Negative**	False Negative (FN)	True Negative (TN)	NPV TN/(TN + FN)
	Sensitivity TP/(TP + FN)	Specificity TN/(TN + FP)	
	Accuracy (TP + TN)/(P + N)		

## Conclusion

Developing a non-invasive method for endometriosis is challenging and currently under investigation. There are new strategies for improving transvaginal ultrasonography skills to diagnose mostly deep infiltrating endometriosis. Biomarker or genetic predisposition studies are being published in a growing manner. Laparoscopy is the most secure way to diagnose endometriosis, but it is an invasive method requiring such that patients should undergo a kind of surgery. Instead, a non-invasive method would be more economical and patient-friendly for the diagnosis of endometriosis. In this respect, as it was demonstrated for the first time in this article, Raman spectroscopy technique together with PCA and the classification algorithms could be a good candidate as a non-invasive diagnostic method for endometriosis.

To further improve this study, one might classify particular spectral bands of the serum spectrum that correspond to the suspected biomarkers of endometriosis. However, because there are insufficient literature data for reference Raman signals of all biomarkers of endometriosis (i.e., annexin V, VEGF, CA-125, slCAM-1^3^), the Raman spectrum of each suspected biomarker should be measured as the reference spectrum to make more reliable inferences about the disease.

## Methods

### Patient Selection

Forty-nine patients who had a surgical diagnoses of endometriosis and 45 healthy women with no history of pelvic pain or infertility were enrolled in this study after ethical approval was granted by the Ethics Committee of the Faculty of Medicine, Acibadem University. Each participant gave written informed consent. All experiments were performed in accordance with relevant guidelines and regulations. Student’s *t*-test was applied on the data of volunteers who joined the study. There were no statistically significant differences between the patient and control groups in terms of age, BMI (body mass index), presence of uterine myomas, and adenomyosis, as given in Table 4. The patients were not divided into subgroups for the investigation because there is no known account to determine whether the main presenting symptom has a different underlying pathophysiology. Four patients had uterine myomas in the patient group, and three women had uterine myomas in the control group; all were asymptomatic. In the patient group, two patients had adenomyosis. Women with comorbidities, drug users, and patients with pelvic pain that was not proven to be endometriosis and who were not on their secretory phase (16–28th day) of the menstrual cycle were excluded.

**Table 4:** Demographic data for the patient and control groups.

	Control group	Patient group	*p*-value
# of Volunteers	45	49	
Adenomyosis (n)	0	2 (4.08%)	0.290
Uterine myoma (n)	3 (6.60%)	5 (10.20%)	0.561
BMI	25.53.3	24.63.6	0.179
Mean Age (years)	27.17.8	29.45.4	0.315

(n): number of patients with myomas/adenomyosis. BMI: body mass index. Confidence level: 0.95.

### Sample Preparation

Blood samples were taken in 10-mL serum separator tube (Vacusera) and centrifuged at 1500 g for 10 minutes to isolate the serum. All the serum samples were stored at 4 °C and measured a maximum of two days after the collection. For the measurement, approximately 0.5 mL of the serum sample was prepared in a quartz cuvette.

### Experimental Setup

**Figure 3:**
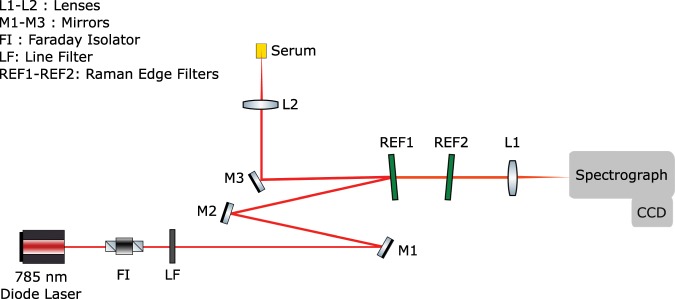
The experimental arrangement for Raman spectroscopy.

The experimental arrangement was built around a home-built microscope that included a water immersion microscope objective (60X, NA, Olympus). A single mode diode laser (CrystaLaser) with wavelength 785 nm and power 100 mW was used for Raman excitation. The unwanted back-reflected beams were filtered using a Faraday isolator (FI, EOTech), which was placed in front of the diode laser. A laser line filter (LF) was employed to obtain a clean laser profile around 785 nm (Semrock, LL01-780-12.5). The sample was illuminated through a focusing lens and the back-scattered light at 180 °C geometry was collected using the same lens. The laser power on the sample was detected around 70 mW. The Rayleigh scattered photons were filtered using two sequentially located Raman edge filters (Semrock). The Raman scattered beam was focused on a 100 *μ*m slit of a spectrometer (f = 303 mm, f#4.3, Andor) using an achromatic lens with a focal length 50 mm. The spectrometer was equipped with a 600 lines/mm grating and with a thermoelectric-cooled CCD camera (at −90 °C, Andor iDus DU420A-OE). A schematic view of the equipment can be seen in Fig. 3.

### Experiment and Analysis

**Figure 4:**
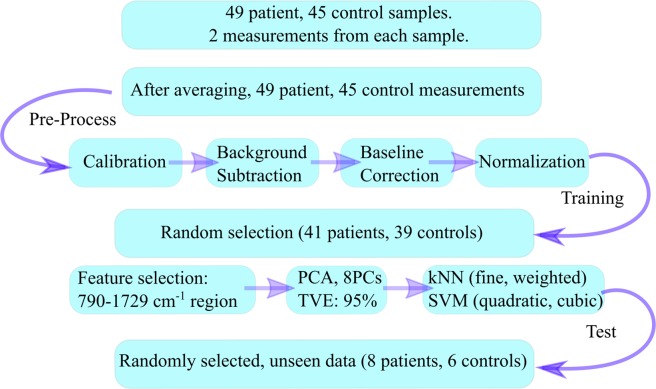
Infographic for pre-processing and the data analysis.

The measurement and spectral analysis scheme is given in Fig. 4. According to this scheme, first, the toluene spectrum was measured using an exposure time of 0.2 s for wavenumber calibration. Secondly, the distilled water spectrum was acquired using an exposure time of 30 s with 14 successive scans. The average of the water spectra was used for the background subtraction. Next, the Raman spectra of the serum samples were measured using the same integration parameters as with the water measurements. Each serum sample was measured twice sequentially. After cosmic-ray removal from the spectral data, 14 scans were decreased to 10 scans by excluding those with higher variance, and then these ten scans were averaged for each measurement. The spectra, which belonged to the same volunteer, were then averaged. Thereby, the data underwent pre-processing through a graphical user interface (GUI) that we wrote on the MATLAB platform. The GUI performs the pre-processing steps, namely calibration, background (BG), and baseline correction (BC), as demonstrated in Fig. 1a. The developed wavenumber calibration method, which uses the Raman spectrum of toluene, was applied^26^. The reference bands of the toluene spectrum were used to calibrate the distilled water and serum spectra^2^. The distilled water spectra were subtracted from the corresponding serum spectra to exclude signals coming from the water and cuvette. This step makes the spectrum background-corrected (BG). After the BG correction, there still remain auto-florescence signals coming from the serum sample. To further exclude these unwanted signals, baseline correction was applied for each spectrum by fitting a cubic spline curve on the selected 12 wavenumber points on the spectrum. To perform baseline subtraction, the selected wavenumbers (corresponding to the data points) were identical for each spline curve to ensure objectivity for each sample. Afterwards, the spline curve was subtracted from the BG spectrum to obtain the baseline-corrected (BC) spectrum (Fig. 1a). Then, vector normalization was applied for each BC spectrum. The mean spectra of the normalized BC data of the two groups can be viewed in Fig. 1b.

To further explore the data, PCA was applied to the vector-normalized BC data. This is a method for data description and compression, which is useful for reducing the dimension of large data sets while preserving most of the information. Its discriminating power for grouping data into clusters makes PCA noteworthy for diagnostic studies. After PCA analysis, built-in MATLAB functions were used to apply kNN (fine and weighted) and SVM (cubic and quadratic) classification methods to construct classification models. The feature selection was performed by re-constructing all the models 10 times for the selected regions because the 5-fold cross-validation algorithm of MATLAB’s classification software is a random process. The standard deviation and the mean accuracy values for each model determined and the best interval of the spectrum, on which the accuracy of classification methods were the highest, were calculated. The average and the standard deviation values of the accuracy calculations are shown in Table 1. After feature selection, 85% of the total spectral data was selected as a training group, which included 41 patient and 39 control measurements. Then, the remaining 15% was set as test data, which contained 8 patient and 6 control measurements. By concerning the training and test results, the specificity, sensitivity, PPV, NPV, and the accuracy of the classification models were calculated according to the equations given in Table 3.

## Data Availability

The corresponding author can provide the datasets of this study upon reasonable request.
